# Some Cases from My Note-Book of Clinical Experience

**Published:** 1910-06

**Authors:** A. W. Fly

**Affiliations:** Galveston, Texas


For Texas Medical Journal.

### Some Cases from My Note-Book of Clinical Experience.

BY A. W. FLY, M. D., GALVESTON, TEXAS.

CASE No. 1.—On September 10, 1898, I was consulted by Mrs. B., a married lady who had no children, for the relief of a floating kidney. She was very much emaciated from constant pain in the renal region. The urine was scant and contained much pus.

I washed out the bladder thoroughly with sterile water. Then had an assistant to press firmly over the line of the ureter for about half an hour, then drew what urine had accumulated in bladder; it was perfectly clear and contained neither pus, blood nor albumen. I was then convinced that I had to deal with a diseased kidney, complicated with renal calculi.

On the morning of September 12th I cut down and lifted the kidney out of the cavity and found it contained a large stone, requiring an incision the whole length of the kidney to remove, and four other stones much smaller, but too large to enter the renal pelvis. The hemorrhage was severe. I at once decided to remove the entire organ, rather than take any chance on secondary bleeding. She promptly recovered, and is today in good health.

CASE No. 2.—About the middle of April, 1894, I was consulted by Mrs. E., mother of one child three years old, with the history that during her pregnancy with this child she suffered with swollen limbs and severe headache the entire time.

The urine showed large amount of albumen and both hyaline granular casts in abundance. Dr. W. C. Fisher was called in consultation, and we soon decided that an abortion was the only thing to do to save her life. We dilated the uterus, plugged with iodoform gauze, removing and replacing same until pains were sufficient to deliver fetus; then under an anesthetic removed the placenta. The recovery was rapid and complete.

About fourteen months later I was again called. At that time she was about two and one-half months pregnant. Dr. Fisher was sent for and we again performed an abortion, as in the first instance, with apparently good results.

The patient from that time seemed fairly in good condition, except headaches and the continuance of the renal trouble.

In the early part of September, 1898, I was again called and found patient suffering intense headache, scant urine that was heavily loaded with albumen and casts. I did not wait for consultation, but at once dilated the uterus and plugged with gauze. The next day Dr. Fisher gave her chloroform and I curetted her thoroughly. She had no unfavorable symptoms afterwards.

On my final visit, I requested her husband to be present instead of the nurse. I submitted three propositions: First, that they occupy separate apartments; second, permit me to remove ovaries; third, change physicians. The second was immediately accepted, with the statement from both parties that they had often wanted the operation done but were afraid to suggest it.

Ten days after curettement I removed both ovaries and tubes. There were no complications. The urine was examined once a month. There was a gradual diminution of all the renal symptoms at each examination. After six months the kidney was apparently normal. The last specimen examined was two years after the removal of the appendages, and all traces of disease had disappeared. Eight years later she died from acute pneumonia.

I have for many years believed that nephritis, both in the pregnant and non-pregnant woman, was in many cases due to a reflex neurosis, and that the removal of the uterine appendages was the radical and rational means of cure.

Case No. 3.—In the early part of May, 1900, I was requested to see an unmarried woman about forty years of age, with a clear history of nephritis of some years standing. Owing to her nervous condition, it was impossible to make an examination without an anesthetic. The physician in attendance objected to the administration of ether or chloroform on account of the renal condition. I assumed all responsibility and he gave ether, under profound anesthesia. The slightest touch over the region of the ovaries caused strenuous contractions of the muscles of the limbs and abdomen. The hymen was intact. Two days later I removed the appendages. She made a good recovery, and today, ten years afterwards, she is a sound and healthy woman without a single trace of kidney disease.

While these two cases by no means are sufficient to settle the question, I think from a clinical standpoint they are worth relating, in order to emphasize to the profession the great importance that reflex neurosis lends to renal disease in the female.

Case No. 4.—Miss S., about 50 years of age, had consulted me on several occasions for what appeared to be a case of renal colic, complaining of severe pain in the kidney and along line of ureter. She had sustained serious injuries during the storm of 1900, and shortly afterwards began to complain from above symptoms.

After long years of suffering, she announced to me that she was ready for the operation, provided I removed the kidney entirely.

The examination of the urine showed large quantity of pus.

On April 6, 1910, assisted by Drs. Moore, Pritchet and Wood, I made a free incision over the line of the kidney. After drawing the organ well out of the cavity, and thoroughly examining it, we failed to find any stone. I then made a free incision through the organ down to the pelvis; found the latter dilated beyond the normal size, then passed a probe down the ureter to the bladder. I then made a free opening over the ureter so that I could trace it from pelvis to bladder. On passing the catheter the urine was bloody, after sewing abdominal wound.

We removed the kidney as promised patient.

Pathological investigation made by Dr. Wood showed inflammation of pelvis of kidney. Had I not promised patient, who was a doctor, before hand, I would have drained the kidney with view of ultimately saving the organ.

Through courtesy of Dr. John T. Moore, this operation was performed in the Christian Sanitarium, Houston, Texas.

The patient seems perfectly well at this time.

Case No. 5.—Compound comminuted fracture of patella (transverse). On September 25, 1909, I was called to see Mrs. S., who was injured while chaperoning an automobile party. The machine was running at a rapid gait and, getting beyond the control of the chauffeur, ran into a drinking fountain located in the middle of the street. All the inmates were thrown to the ground in a violent manner.

I found Mrs. S. badly contused over the entire back and limbs. The right patella was completely crushed and the ligamentum patellæ was severed. I made a free vertical incision through the joint, removed a large number of bone fragments, then brought the two ends of the patella together by boring a hole in each fragment and approximating the same with silkworm gut. I sewed the periosteum with 30-day acromized catgut, made counter openings on either side of the joint, and inserted medium-size drainage tubes; stitched external wound with silkworm gut and placed the limb in Lever's splint. I dressed the wound at first every day, then every other day, until it was healed. The limb was kept immovable for six weeks. Then passing motion was made at regular intervals for three months.

There has been no limping, and the use of the limb is perfect.

